# The New Wave of Gene and Cell Therapies Across Diseases

**DOI:** 10.3390/jcm15051799

**Published:** 2026-02-27

**Authors:** Adrianna Rieske, Dagmara Grot, Cezary Tręda, Aneta Włodarczyk, Ewelina Stoczyńska-Fidelus, Maria Jaskólska, Piotr Rieske

**Affiliations:** 1 Department of Research and Development, Personather Ltd., Inwestycyjna 7 St, 95-050 Konstantynow Lodzki, Poland; adrianna.rieske.25@ucl.ac.uk (A.R.); cezary.treda@umed.lodz.pl (C.T.); aneta.wlodarczyk@umed.lodz.pl (A.W.); piotr.rieske@umed.lodz.pl (P.R.); 2 Global Business School for Health, Faculty of Population Health Sciences, University College of London, Gower Street, London WC1E 6BT, UK; 3 Department of Tumor Biology, Medical University of Lodz, Zeligowskiego 7/9, 90-752 Lodz, Poland; maria.jaskolska@student.umed.lodz.pl; 4 Department of Molecular Biology, Medical University of Lodz, Zeligowskiego 7/9, 90-752 Lodz, Poland; ewelina.stoczynska-fidelus@umed.lodz.pl

**Keywords:** gene therapies, cell therapies, CAR-T, CRISPR, genetic engineering

## Abstract

Recent years have seen rapid progress in biological treatments for genetic diseases, as well as conditions like type 1 diabetes that lack an obvious genetic component. The authors sought to explain why this progress has emerged at this particular moment. The best way to illustrate this is by showcasing a wide range of therapies targeting diverse diseases. This progress has been driven by technological advances in genetically modified CAR-T and CAR-NK cells (e.g., using CRISPR or transgenes), which have led to significant improvements in cancer therapy. A key trend now is the emergence of “off-the-shelf” approaches aimed at generating cellular therapies compatible with a range of recipients by mitigating alloreactivity and immune rejection. Different diseases impose distinct biological and logistical limitations; thus, treatment of each patient requires an appropriate strategy. Emerging advances include the modification of therapeutic cells, either ex vivo or in vivo. Current options for transgene delivery mainly comprise lipid nanoparticles (LNPs), adeno-associated virus (AAV) vectors, and lentiviral vectors. Researchers also focus on selecting suitable promoters for specific expression in selected cell types. Altogether, these advances have led to remarkable progress in treating various diseases in recent years. This publication discusses the development of biological therapies, with particular emphasis on cell and gene therapies, illustrated by viable examples across various disorders. It covers implemented solutions for several types of cancer, as well as selected hereditary diseases and syndromes, including Huntington’s disease, carbamoyl phosphate synthetase 1 (CPS1) deficiency, hemiplegia, epidermolysis bullosa, chronic granulomatous disease, and congenital deafness. Emerging applications in heart diseases and diabetes are also summarized, along with therapeutic strategies involving tRNA gene editing. Although numerous strategies exist, only the most representative, practical, and up-to-date examples are emphasized.

## 1. Introduction

Although gene and cell therapies long fell short of expectations, significant progress has been made in recent years. Kohn et al., in their article, present both successful and less effective examples of therapies and aim to reflect on why such a positive shift has become apparent only now [1]. To illustrate the drivers of this change, we begin the review with disease areas in which direct cell administration remains central to therapeutic efficacy. Therefore, the first part discusses examples of approaches in which cell administration is currently considered crucial—for instance, in the treatment of type 1 diabetes or Parkinson’s disease. In these cases, patient-specific cell therapies are being developed. These cells can undergo ex vivo genetic modifications to achieve selected traits [2,3].

The results of work on the development and improvement of transgene delivery systems increasingly enable solutions in which cell collection and ex vivo procedures are no longer required [4]. Therefore, the second part of the article discusses in vivo approaches that serve as an alternative to generating therapeutic cells outside the patient’s body, including in the context of genetic disorders.

The third part discusses cell therapies that use modified cells engineered to express synthetic genes, such as CAR (e.g., CAR-T cells). Synthetic biologists are working to design an increasingly broad repertoire of such constructs [5]. In this context, both in vivo and ex vivo approaches are explored. Gradually, a paradigm shift is taking place—a move away from strictly ex vivo therapies in favor of in vivo approaches [6]. On the other hand, ex vivo strategies still allow for more extensive and controllable cellular modifications since comprehensive long-term safety evaluations of effects, including mutagenesis, immune-mediated reactions, and carcinogenesis of in vivo approaches, may only become evident after extended periods of observation.

Two main strategies can also be observed: one focuses on less extensively modified immune cells with CAR transgene only (e.g., CAR-NKT cells), while the other explores progressively broader genetic alterations of therapeutic cells. Certainly, the presented examples do not exhaust all recent achievements in the field; however, in the authors’ opinion, it represents an important and illustrative selection.

Why has the breakthrough occurred precisely now?

The current breakthrough in gene and cell therapies, particularly evident between 2023 and 2026, results from the convergence of several key technological advances that have overcome long-standing limitations in delivery, precision, scalability, and safety. By presenting a broad range of therapies for different patient populations across a wide spectrum of diseases, it is possible to explain this phenomenon. The acceleration of lipid nanoparticle (LNP) technologies—initiated by the success of mRNA COVID-19 vaccines—has enabled efficient in vivo delivery of transgenes and gene-editing tools to organs such as the liver and to cells of the immune system. These advances have enabled a transition from complex ex vivo procedures to simpler, more cost-effective, and more accessible in vivo applications, significantly reducing both the cost and logistical burden of therapy [7]. At the same time, improvements in CRISPR-derived technologies—such as base editing (ABE) and prime editing—have enabled safer and more precise genetic modifications without inducing double-strand DNA breaks. This has opened the door to therapies for rare diseases, including suppressor tRNA editing for nonsense mutations [8] and correction of mutations in genes such as *NCF1* in chronic granulomatous disease [9]. Hypoimmunogenic engineering—based on the disruption of *B2M* and *CIITA* genes combined with the overexpression of *CD47*—has overcome immune rejection, enabling the development of universal, off-the-shelf allogeneic therapies. Examples include hypoimmunogenic CAR-NK cells for systemic sclerosis [10] and pancreatic islet transplantation in type 1 diabetes [11]. Finally, tools from synthetic biology, including tissue-specific promoters (e.g., *Myo15* in otoferlin-related deafness therapy, where the transgene is delivered via dual AAV without CRISPR editing [12], and *GfaABC1D* in in vivo CAR expression in astrocytes [13]), as well as multigene modifications, have extended these approaches to complex tissues such as the central nervous system and the inner ear. The synergy of these elements—previously absent due to limitations in delivery platforms, editing fidelity, and immunogenicity—has driven a paradigm shift from autologous ex vivo therapies toward more accessible in vivo and universal strategies, thereby opening the path to broader clinical application [1,6,7,14]. Importantly, in 2025, the FDA approved a record number of gene therapies (more than ten), including label expansions for existing products such as Zolgensma, underscoring the maturity of the field and growing regulatory confidence [7]. Nevertheless, challenges such as off-target effects, durability of expression, manufacturing costs, and the risk of immunogenicity associated with novel editors continue to require further innovation, including the use of artificial intelligence to optimize vectors and editing strategies [14].

## 2. Typical Ex Vivo Cell Therapies, Excluding CAR-T

**Figure 1:**
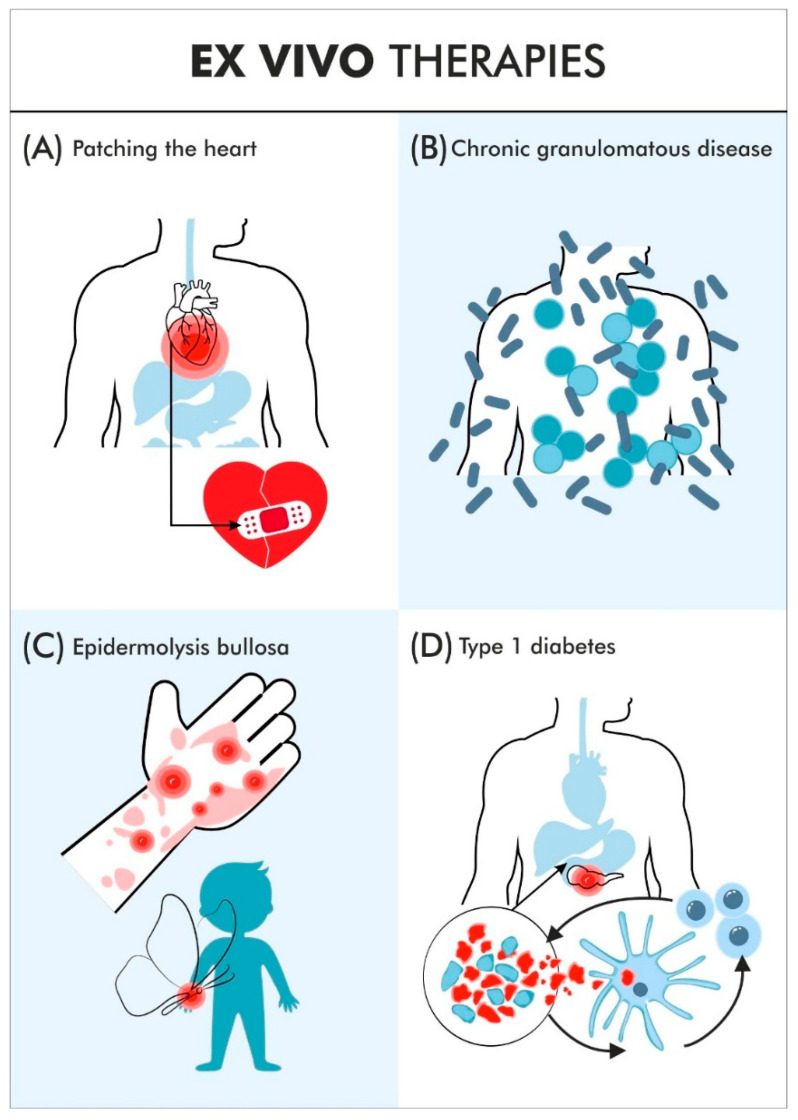
The article describes several examples of ex vivo cell therapies. Three of them clearly also qualify as gene therapies. The exception is example (**A**), in which iPSCs were used to generate cardiomyocytes. Reprogramming alone does not fully meet the criteria for gene therapy, even though transgenes are used in the process. Example (**B**) concerns editing of the gene responsible for chronic granulomatous disease. The edit was performed in HSCs using CRISPR technology. Example (**C**) involves introducing a collagen transgene into patients’ keratinocytes/fibroblasts for the treatment of epidermolysis bullosa, using a retroviral vector. Example (**D**) relates to therapy for individuals with type 1 diabetes. The publication describes two approaches. One involves generating insulin-producing cells from ESCs for different recipients; this strategy requires immunosuppression. The second approach is based on modifying pancreatic cells from deceased donors using CRISPR and introducing a CD47 transgene so that the transplanted cells are not rejected in an allogeneic setting.

The first diseases in which gene and cell therapies have been successfully applied in recent years are sickle cell disease and β-thalassemia. The erythroid-specific enhancer region of BCL11A in hematopoietic stem cells was modified using the CRISPR/Cas9. After transplantation, the defective erythrocytes were rapidly replaced by corrected erythrocytes with improved hemoglobin composition. The circumstances were relatively favorable because hematopoietic stem cells could be modified ex vivo [15]. Similar ex vivo strategies can be applied to various genetic disorders affecting blood or bone marrow cells. In the case of BEAM-101 therapy for sickle cell disease, instead of CRISPR/Cas9, a precise base editing technique (specifically A-to-G base editing) is used [16]. A related example is ex vivo gene therapy using a lentiviral vector to introduce a modified β-globin gene (βT87Q-globin) into the patient’s hematopoietic stem cells. The therapy was approved by the FDA in December 2023 [17]. Another example of an ex vivo developed therapy is the treatment of patients with Wiskott–Aldrich syndrome (WAS), a rare, X-linked genetic disorder, characterized by a classic triad of immunodeficiency caused by a mutation in the WAS gene. Using a lentiviral vector carrying WAS cDNA, corrected protein production was achieved in various cell types derived from hematopoietic stem/progenitor cells (CD34^+^) [18]. In some cases, therapeutic success depends on the classical administration of cells rather than gene correction. In one example, cardiomyocytes derived from induced pluripotent stem cells (iPSCs) were used (Figure 1A). This was not an autologous transplant and represents one of the few examples in which allogeneic therapy was chosen. The treatment was used as a temporary solution to “patch up” the heart and stabilize cardiac function, allowing the patient to survive until a heart transplantation can be performed. Preclinical studies in macaques were successful and led to approval for the temporary use of cell therapy in a woman awaiting heart transplantation (bridge-to-transplant). Post-transplantation analysis of the “patched” heart confirmed a positive effect of the cell therapy. However, immune-cell infiltration was also detected, indicating that this approach is likely to remain a temporary solution rather than a long-term effective therapy using allogeneic cardiac cells [19].

Another situation in which cell therapy appears feasible without introducing genetic modification into these cells involves defects in microglia. According to Wu et al., such conditions can be referred to as microgliopathies [20]. Surprisingly positive therapeutic effects have been observed in all cases where healthy microglial cells acted beneficially within the central nervous system (CNS). In this example, the therapy concerned the brain disorder called Adult-Onset Leukoencephalopathy with Axonal Spheroids and Pigmented Glia associated with mutations in the CSF1R gene. In the CNS, the defective CSF1R protein is abundantly expressed (with high membrane density) in microglial cells. Mutations in both alleles of this gene are perinatally lethal, while a mutation in a single allele leads to a rare, essentially fatal disorder—a “microgliopathy”. Therefore, a large proportion of the patient’s defective microglial cells were replaced. To achieve this, it was necessary to ablate the patient’s bone marrow and perform a transplant from a healthy donor. Over time, the affected cells in the central nervous system (CNS) underwent cell death and were replaced by corrected donor-derived microglial cells. In the past, attempts had been made to treat these patients using iPSCs in which the pathogenic mutation had been corrected with CRISPR technology. Now, according to Chadarevian et al., autologous transplantation of CSF1R-corrected microglia is not the only option; allogeneic CSF1R-wildtype iPSC-derived microglia can also reduce the diverse neuropathologies found in ALSP, without requiring the use of corrective CRISPR editing [21]. Mader et al. argue that the rescuing transplant can be allogeneic in part because the central nervous system (CNS) exhibits a certain degree of immunological tolerance. Nevertheless, immunosuppression may still be necessary [22].

Implementation of bone marrow transplantation-based therapy also had a significant impact on positive long-term effects in patients with childhood-onset cerebral X-linked adrenoleukodystrophy. Before this therapeutic approach became available, patients typically survived only three to five years after the onset of clear symptoms [23]. If autologous bone marrow transplantation can be life-saving in certain CNS disorders, this represents a major advancement [24].

An analogous approach has been applied in the treatment of type 1 diabetes, whereby the administered cells were not genetically modified (Figure 1D). Patients received islet-like cell surrogates derived from embryonic stem cells. In 10 out of 12 patients, the therapy yielded substantial clinical improvement. However, it remains unclear how durable the therapeutic effect will be, as this strategy does not address the underlying cause of the disease, which is the destruction of pancreatic islets by the patient’s own autoreactive cytotoxic lymphocytes. After one year of observation, however, the patients’ conditions remained stable, although they required maintenance of immunosuppressive therapy. Despite significant advances, differentiating stem cells into pancreatic cells is one of the most challenging processes [25]. An alternative approach involves harvesting cells from a deceased donor and subsequently making them “universal.” Donislecel, an allogeneic but non-modified pancreatic islet cell therapy derived from deceased donors, was designed to replace destroyed insulin-producing β cells in adult patients with type 1 diabetes. In June 2023, it was approved by the FDA as the first cell-based therapy for type 1 diabetes in adults who, despite intensive insulin therapy, experience severe hypoglycemic episodes and have difficulty achieving target HbA1c levels. The mechanism of action involves the infusion of pancreatic islet cells into the hepatic portal vein, allowing them to engraft in the liver and secrete insulin in a physiologic manner. In some patients, this approach may reduce or even eliminate the need for continuous exogenous insulin administration [26]. In another project, complex genetic modifications were introduced to prevent the destruction of these cells by the patient’s immune system. Interestingly, the cells were implanted into muscle tissue because pancreatic surgeries required for direct cell implantation are technically difficult. Interestingly, immunosuppression was not required, although this was an early-stage study involving a single patient. Using CRISPR technology, two HLA genes were knocked out in the donor cells to prevent T-cell–mediated recognition: B2M (responsible for MHC I presentation) and CIITA (responsible for MHC II). However, cells lacking B2M and CIITA were still destroyed by natural killer (NK) cells and macrophages. To overcome this, an additional CD47 transgene was introduced into the donor cells using a lentiviral vector. Only cells combining knockout of B2M and CIITA with CD47 overexpression were protected from destruction by any components of the recipient’s immune system. Following transplantation, diabetes-related parameters improved and stabilized, although insulin administration was not discontinued, but its dosage was reduced [11]. Pancreatic surgeries required for direct cell implantation are technically challenging, which reduces the therapeutic potential (for instance, due to a lack of response to paracrine intestinal hormones), but does not eliminate it. Importantly, all genetic modifications were performed ex vivo, and in the reported case, genetic alterations by lentiviral vector in oncological CAR-T therapies are classified as a precedent for safety [27,28]. Another team conducted similar preclinical research and successfully differentiated iPSCs into cells capable of producing and releasing insulin properly [29]. Similar genetic modifications were also performed in pancreatic cells [30]. This model will also be discussed in the context of CAR-T cells. Alternatively, CD4+ CD25highCD127 Treg-based therapy offers a promising immunotherapeutic strategy for type 1 diabetes patients. The Tregs+antiCD20 patient group was superior to the Standard of Care control group in terms of C-peptide secretion in the mixed-meal tolerance test, the time of insulin independence, and remission [31].

**Figure 2:**
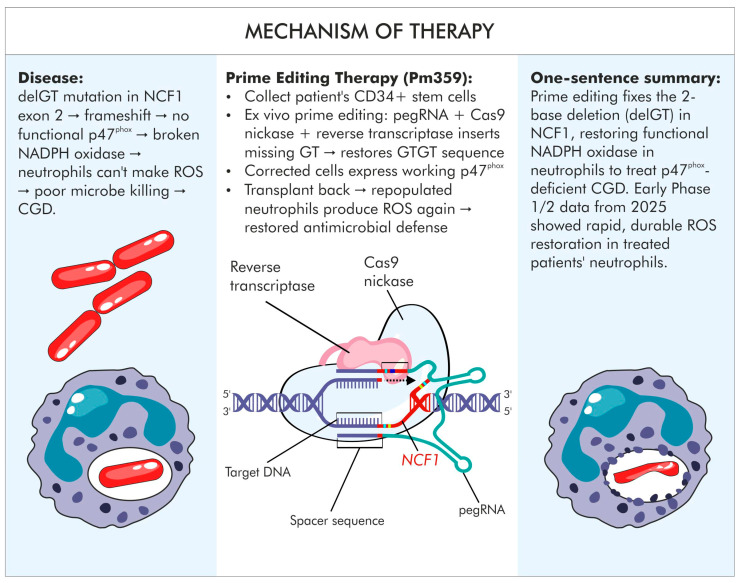
Correction of the NCF1 gene mutation in chronic granulomatous disease using prime editing. The scheme depicts the organization of the NCF1 gene and its pseudogenes (NCF1B and NCF1C) in a healthy individual and in a patient with p47^phox^ deficiency. The mechanism of restoring the correct NCF1 reading frame, leading to reconstitution of a functional NADPH oxidase complex and recovery of reactive oxygen species production in neutrophils, is illustrated. The infographic was prepared based on Jennifer L. Gori et al., 2025 [32].

Another example of ex vivo therapy application is chronic granulomatous disease (CGD) (Figure 1B and Figure 2). The most common cause of CGD is a two-nucleotide deletion (delGT) in the Neutrophil Cytosolic Factor 1 (NCF1) gene encoding the p47^phox^ protein. Worth noticing is the complex genetic context of NCF1, which is flanked by highly homologous pseudogenes (NCF1B and NCF1C) naturally carrying the delGT variant (Figure 2) [32]. The absence of p47^phox^ impairs host defense against pathogens, primarily bacteria [33]. CDG occurs with a frequency of approximately 1 in 150,000 births. The therapeutic approach involved autologous hematopoietic stem cells (HSCs) edited ex vivo using prime editing to restore the correct GTGT reading frame. This represents the first therapeutic use of prime editing, targeting the pathogenic delGT mutation in the NCF1 gene. A single dose of intravenously administered modified HSCs restored NADPH oxidase activity in 66% of neutrophils (Figure 2) [9].

Other solutions have been proposed for recessive dystrophic epidermolysis bullosa (RDEB), a particularly severe and difficult-to-treat form of epidermolysis bullosa (Figure 1C). The skin of affected patients is sometimes compared to the delicate wings of a butterfly. Patient-derived cells obtained by biopsy were transduced with elements from a replication-deficient retroviral vector carrying the cDNA of the gene encoding type VII collagen (COL7A1), which is mutated in affected individuals. Next, the patients’ wounds were covered with skin grafts composed of their own modified cells [34]. Among 43 wounds treated with a single application, 81% showed at least a 50% improvement in healing compared to approximately 15% of 43 matched control wounds treated with standard therapy (assessed after six months). In seven patients with a total of 38 wounds, a single surgical application of the therapeutic product resulted in long-term improvement at the treated sites, lasting on average nearly seven years.

## 3. Gene Therapies Without the Need for Ex Vivo Cell Modification

**Figure 3:**
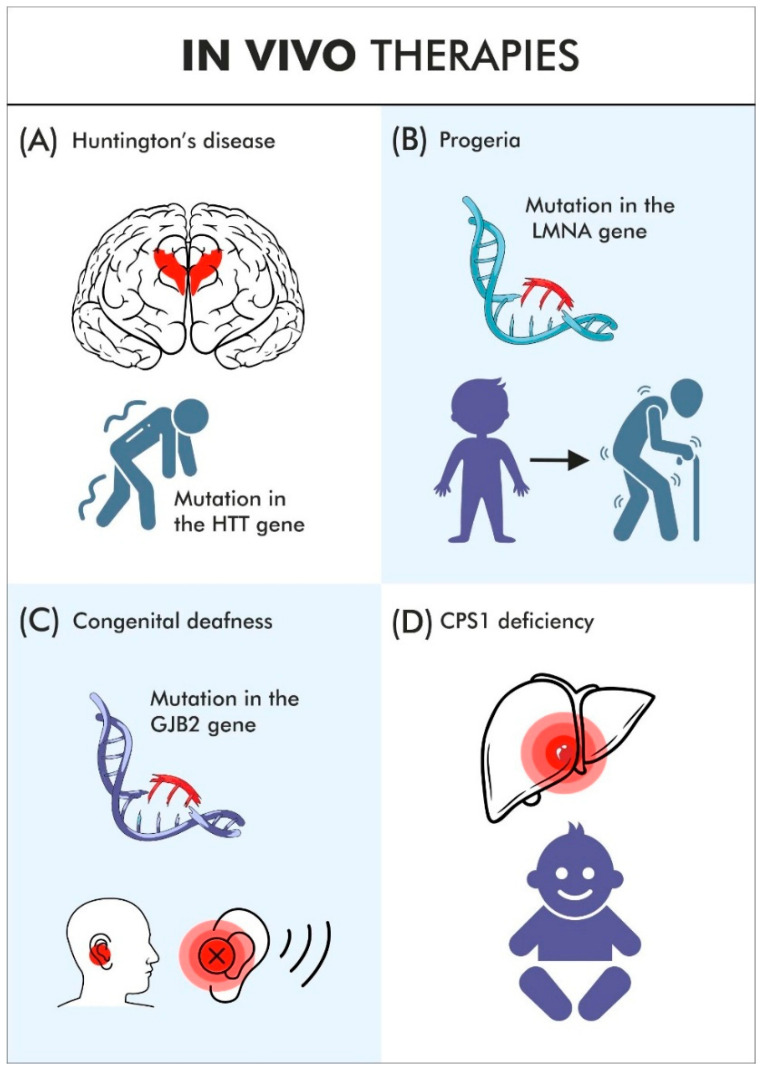
The article describes several examples of in vivo therapies. (**A**) Huntington’s disease. An miRNA was used that suppresses both the wild-type huntingtin mRNA and the mutant, aggregation-prone form. The AAV vector was delivered to specific CNS structures under MRI guidance. (**B**) Progeria. Cas13 was used to eliminate progerin mRNA, which is defective due to a silent mutation that causes aberrant splicing. Cas13 and its gRNA were delivered into human fibroblasts using a lentiviral vector. Systemic studies have so far been conducted only in animal models. (**C**) The described form of congenital deafness caused by an otoferlin mutation. Two vectors encoding different portions of the gene’s cDNA are introduced. In addition, an appropriate promoter (Myo15) is used to ensure transgene expression in the correct hair-cell population. Two cooperating AAV vectors are required because of the size of the transgene. (**D**) In diseases caused by defects in liver cells, various therapies based on LNPs and genome editing are being developed. Metabolic disorders—such as carbamoyl phosphate transcarbamylase deficiency—are at the forefront of these efforts.

Recently, therapeutic approaches have emerged for more complex cases in which treatment does not necessarily require ex vivo cell modification. Notably, early clinical studies have already shown efficient transduction with sustained CAR expression and initial signs of antitumor activity due to T-cell–tropic lentiviral vectors and LNP delivering CAR-encoding mRNA in vivo. This aims to facilitate manufacturing, reduce costs, and simplify complex logistics [35]. For example, progress has been made in developing therapies for certain genetic disorders of the CNS. One such condition is alternating hemiplegia of childhood (AHC), which affects approximately one child per million [36,37]. The disease is caused by mutations in the ATP1A3 gene, which encodes the α3 subunit of the Na^+^/K^+^-ATPase transmembrane ion pump, causing highly complex neurological symptoms [38]. In a mouse model, postnatal AAV9-mediated correction of pathogenic ATP1A3 variants in the CNS was successful in approximately 50% of cortical neurons. AAV viral vectors are episomal; thus, they do not replicate with cell division, but still create a clinically important phenotype and extend lifespan [37]. A particularly striking case involved a boy with a rare metabolic disorder associated with liver dysfunction—severe carbamoyl phosphate synthetase 1 (CPS1) deficiency [39] (Figure 3D). Previously, liver transplantation was a standard therapy for children with carbamoyl phosphate synthetase 1 (CPS1) deficiency or related urea cycle disorders [40]. Without transplantation, death was common; even when a donor was found, severe brain damage often occurred beforehand [1,41]. Genomic sequencing showed compound heterozygous CPS1 deficiency (one pathogenic allele inherited from each parent—Q335X inherited from the father and E714X inherited from the mother); thus, the research team selected a CRISPR-based-editing paternal variant approach rather than a double-strand-break nuclease strategy [42]. To optimize the therapy for safety and efficacy, researchers created both a cellular model mimicking the patient’s altered human hepatocytes and an animal model replicating the boy’s genetic mutations. During this period—spanning several months—clinicians were fighting to keep the child alive. These models were used to test different gRNAs and select optimal PAM sequences, refining the therapeutic design to ensure efficient correction at the mutation sites while minimizing off-target effects [43].

Instead of correcting cells ex vivo, researchers employed LNPs to encapsulate the mRNA therapeutic molecules for intravenous administration. The LNPs were specifically engineered and delivered in a way that ensured predominant accumulation in the liver. In animal studies, the gene correction rate reached as high as 40% of hepatocytes, demonstrating significant therapeutic potential.

In liver diseases, the situation is somewhat more favorable than in CNS disorders, because after intravenous administration, LNPs predominantly accumulate in the liver [44]. From this perspective, the application of this therapy was, therefore, easier than in the previously described case of alternating hemiplegia.

Importantly, this patient will not be the only beneficiary. The therapeutic strategy developed for him may be applied to other children with genetic liver diseases, and potentially to disorders affecting other organs as well. A key aspect of this breakthrough is that it represents an in vivo therapy enabled by lipid nanoparticle delivery, rather than an ex vivo approach involving modification of stem cells outside the patient’s body followed by reinfusion. Reflecting the growing promise of such personalized in vivo gene editing approaches, the FDA has recently approved the introduction of therapies based on this approach for several hepatocyte-related genetic diseases [45,46].

Moreover, the use of genome editing in vivo is being considered in patients with hypercholesterolaemia, not only in those with the familial form of the disease. Researchers have disrupted the ANGPTL3 gene in hepatocytes using CRISPR/Cas9 and mRNA lipid nanoparticles in order to lower levels of “bad cholesterol” (LDL). Individuals who naturally have reduced activity of this gene are less likely to develop LDL-related disease. Results from the first phase of clinical trials conducted in a group of several dozen patients have been published [47]. The LNPs were additionally tuned to bind the LDL receptor and scavenger receptors on hepatocytes (in the liver) and to be internalized in a manner similar to LDL. So far, this approach has been used only in seriously ill patients. However, not all of them had specific hereditary diseases or syndromes. This suggests that the boundary between treatment, prevention, and enhancement may gradually begin to blur. At present, the liver has become an especially intensively studied organ because of its high susceptibility to nanoparticle internalization. Aside from the elimination of HLA genes to produce universal CAR-T or CAR-NK cells, this is the only case in which a normal (non-mutated) gene has been deliberately inactivated [47].

Another significant advance in in vivo therapies involved the treatment of progeria (Figure 3B). This represented a different application of CRISPR-based technology—this time used not to repair or remove a gene, but to target and destroy the mRNA (Cas13 instead of Cas9) encoding the protein responsible for the disease. The disorder, known as Hutchinson–Gilford progeria syndrome (HGPS), is extremely severe and typically fatal within the first two decades of life. It affects approximately one child per one to four million births [48,49]. In the particular form of progeria for which this therapy shows promise, the disease is caused by a mutation in the lamin A gene (LMNA) [50]. Interestingly, in this case, a lentiviral vector was used in vivo. This was necessary because the vector needed to reach dividing cells. By contrast, in ex vivo CAR-T cell therapy—which will be discussed in the following section—the use of an integrating viral vector is more acceptable, as it is applied only to isolated cells outside the patient’s body. Here, however, various cells in the organism were deliberately exposed to the vector, which raises safety concerns. This issue is also a topic of ongoing debate in the context of in vivo CAR-T therapies. The mutation targeted in this approach does not alter an amino acid and might appear “silent.” Nevertheless, it disrupts mRNA splicing, leading to the production of defective forms of lamin A. The therapy enables the selective degradation of abnormal transcripts. In this case, the use of Cas13 was required [48]. The studies were conducted in mouse models. Importantly, this therapeutic concept extends beyond progeria. It is estimated that at least 10% of genetic diseases and syndromes result from the presence of defective mRNA molecules—often caused by aberrant splicing, as in this variant of progeria [38,50].

A particularly interesting combination of gene therapy in vivo and neurosurgery was implemented at University College London (UCL) in patients with Huntington’s disease (Figure 3A). An AAV vector carrying DNA encoding a microRNA (miRNA) designed to degrade mRNA transcripts of both the normal and mutant forms of huntingtin was delivered directly into specific structures of the central nervous system (CNS) using a specialized catheter system under MRI guidance. It is estimated that the progression of the disease was significantly slowed—a degenerative process that typically advances over about one year was delayed to approximately four years following treatment [51].

Another example is the gene therapy for spinal muscular atrophy approved by the FDA in November 2025. The therapy is based on an AAV9 vector that delivers a functional copy of the SMN1 gene to motor neurons and other target cells, restoring SMN protein production [52]. Promising results have also been reported from clinical trials of gene therapy for one form of congenital deafness caused by mutations in the *OTOFERLIN* gene. According to the authors, this gene therapy may offer advantages over cochlear implants for this particular type of deafness [12].

There are numerous genetic causes of hearing loss—in the United States, approximately 1 in 1000 children is affected. Mutations in *OTOFERLIN* account for about 1–3% of congenital deafness cases (Figure 3C). The therapeutic approach employed an AAV viral vector in collaboration with surgical teams. In this case, two vectors were required to generate such a large mRNA fragment encoding otoferlin. The transgene fragments delivered by the two cooperating vectors were joined through the SD-AP and AP-SA sequences. These sequences facilitate transcription and promote recombination, resembling splicing [53]. Together, these examples illustrate that CRISPR-based methods are not always the superior solution. Each mutation requires a separate gRNA. In contrast to genome-editing approaches, the introduction of a therapeutic transgene allowed for the correction of multiple distinct mutations simultaneously. The disease is autosomal recessive. Moreover, the therapeutic transgene was expressed primarily in the hair cells of the organ of Corti, which enhanced both efficacy and safety. To achieve selective expression, the Myo15 promoter was used. Only one child in the trial did not show improvement. In some patients, cochlear implants were deactivated following successful gene therapy. Remarkably, hearing improvement was observed even in a 16-year-old patient. As in the case of carbamoyl phosphate synthetase 1 deficiency, researchers considered the possibility of an immune response against the newly produced therapeutic protein, which the patient’s immune system might recognize as foreign. However, no clinically significant immune reactions have been observed so far [12].

In contrast to gene editing, gene therapies based on transgene delivery require considering the sizes of the genes. Duchenne muscular dystrophy (DMD) micro-dystrophin constructs (typically shortened to ~3.5–4.4 kb, compared with the full-length dystrophin of ~11–14 kb) represent the most well-known example of a disease in which the transgene had to be radically truncated to fit within the packaging capacity of AAV vectors (Table 1). Ongoing studies conducted within programs such as Elevidys (Sarepta) and RGX-202 (REGENXBIO) demonstrate that this strategy can be therapeutically effective.

Gene therapies targeting large genes inherently require compromises: micro-versions of the transgene lose some functional domains but retain sufficient activity to produce clinical benefit. In the case of DMD, the spectrum of mutations observed to date has not yet been particularly amenable to genome-editing approaches, making transgene replacement strategies the preferred option at present [54].

A very interesting approach has been developed by the team led by Suki Albers in the therapy of several rare diseases. The prerequisite is the presence of mutations that introduce a premature STOP codon. They modified the tRNA that normally recognizes the codon for an amino acid so that it recognizes the STOP codon instead. In theory, this approach has been explored for a long time [55]. However, earlier attempts introduced a transgene encoding such a tRNA using AAV or lentiviral vectors [56]. In this approach, the authors proposed modifying the tRNA gene itself using a specialized variant of prime editing.

## 4. CAR-T Cell Immunotherapies (Including CAR-NK and CAR-NKT)—In Vivo and Ex Vivo Approaches, Genetic Modifications, and Cell Selection

In the field of CAR-T cell therapies, remarkable progress has been achieved in recent years, with currently approved CAR products being predominantly autologous ex vivo CAR-T therapies (Table 2). One of the major challenges now is to make such treatments available “off-the-shelf.” Several strategies are being pursued toward this goal [57].

The first strategy involves genetic modification of CAR-T cells in a manner similar to that described earlier for pancreatic β-cells—introducing changes that allow these therapeutic cells to be used in any recipient.

In a notable study, Hypoimmune CD19 CAR-T cells were engineered to evade allorejection in patients with cancer and autoimmune diseases [58]. The approach was conceptually similar to that used by the same team in their research on pancreatic cells, which were derived not from deceased donors but from induced pluripotent stem cells (iPSCs). The HIP-engineered primary CD19 CAR-T cells required three major genetic modifications to ensure effective function and prevent graft rejection. Undoubtedly, insertion of the CAR transgene was essential in this case. As discussed later, other research groups have introduced additional modifications to further improve the performance and controllability of CAR-T cells.

Another approach to achieving universal applicability—not mutually exclusive with the first—involves the selection of alternative immune cell types, such as CAR-NKT cells. This concept has since been extended to other immune cell subtypes.

Finally, there is the emerging possibility of applying CAR-T therapies in vivo. A research team successfully generated CAR-NK cells compatible with any recipient. These cells carried as many as nine genetic modifications in total: three genes were deleted, four natural transgenes were introduced, and two synthetic genes were added. The researchers derived their CAR-NK cells from iPSCs, which are considerably easier to modify genetically than CAR-NK cells isolated directly from donors—and, for many research groups, easier still than modifying pancreatic cells as described earlier [10].

The CAR-NK cells, however, required a greater number of genetic modifications, as they were designed to enable controlled activation and targeting. By contrast, fewer modifications were needed in the pancreatic cell models described earlier, although pancreatic cells are far more difficult to derive from iPSCs than CAR-NK cells.

Interestingly, these CAR-NK cells were not used to treat an oncological patient, as is often the case with CAR-T or CAR-NK therapies, but rather a patient suffering from an autoimmune disease—systemic sclerosis (scleroderma). This condition is characterized by fibrosis of the skin and, in some cases, fibrosis of internal organs [59]. Although systemic sclerosis today less frequently leads to death, the selected patient was a critical case, and the study also highlighted the broader potential of such genetic and epigenetic cell modifications. The researchers used a variant of CRISPR technology to perform precise gene edits. Specifically, they knocked out *B2M*, *CIITA*, and *CD16* while introducing two HLA genes to make the cells universal. They further enhanced the cells with IL-2RF to boost therapeutic efficacy and added tEGFR as a safety switch, allowing physicians to mitigate potential complications if needed. Additionally, they inserted two CAR transgenes targeting BCMA and CD19 [10].

Much recent attention has focused on the concept of in vivo CAR-T generation within the patient’s body. This is a highly promising strategy—discussed later—but the extent of genetic modification achievable in vivo remains far more limited than that possible ex vivo.

An intriguing alternative to cancer immunotherapy involves the use of CAR-NKT cells, which can be developed as universal therapeutic products. Preclinical studies have been conducted in ovarian cancer, a disease that remains a major medical challenge but may benefit substantially from this approach [60].

NKT cells are relatively easy to derive for any recipient (compared with CD8 cytotoxic lymphocytes). They represent a natural “chimera” of NK and T lymphocytes, serving as a potential alternative and competitor to the universal CAR-NK approach developed in China. Chinese researchers achieved broad compatibility by introducing nine genetic modifications into CAR-NK cells.

CAR-NKT cells are based on naturally occurring immune cells, which require fewer genetic alterations to achieve universality. Moreover, these engineered CAR-NKT cells were found to modulate the tumor microenvironment, making it more favorable for tumor cell destruction. These results were obtained in preclinical studies.

A very interesting approach is presented in the work by Chiesa et al. [61].

Three key genes were edited to address the main challenges associated with using CAR-T cells targeting CD7 in patients with T-ALL. Knockout of the CD7 gene prevents CAR-T cells from self-reactivity (fratricide). Knockout of CD52 renders the cells resistant to alemtuzumab, an anti-CD52 antibody used during lymphodepletion. Finally, knockout of TRAC (TCRα chain) eliminates the risk of graft-versus-host disease (GVHD) [61].

**Figure 4:**
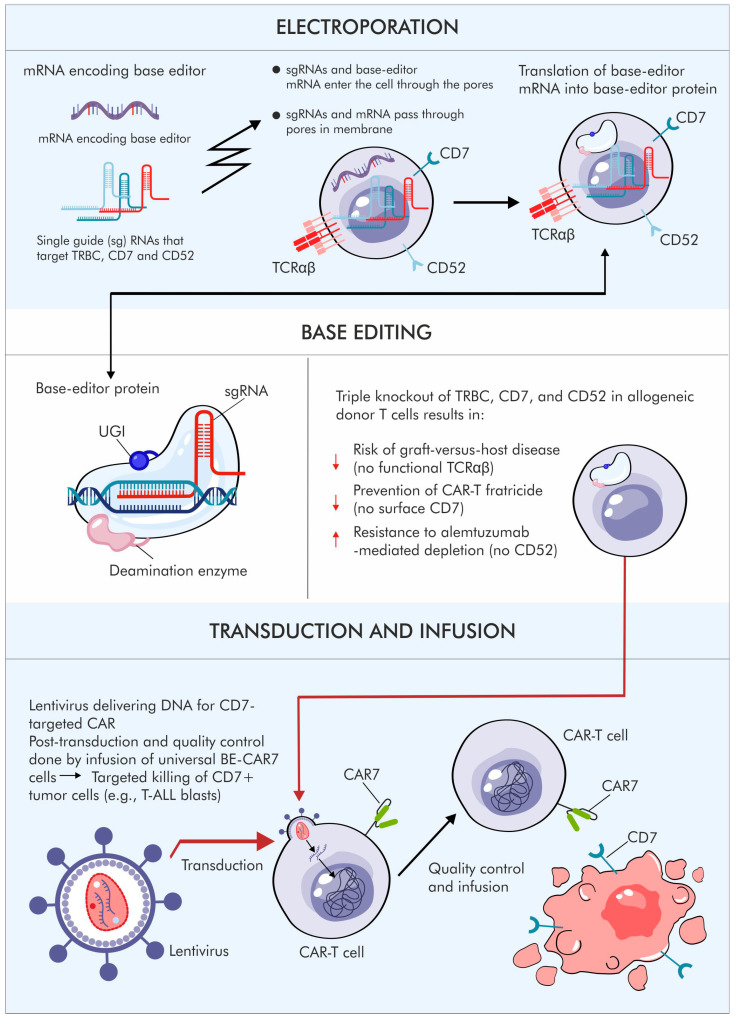
Generation of allogeneic CAR-T lymphocytes using base editing in the therapy of hematological malignancies. The scheme illustrates the process of manufacturing allogeneic CAR-T cells, encompassing electroporation of mRNA encoding the base editor and sgRNA, selective inactivation of the TRBC, CD7, and CD52 genes via base editing, and subsequent transduction with a lentiviral vector encoding the CAR receptor. The modified CAR-T cells exhibit reduced risk of graft-versus-host disease, limited fratricide among T lymphocytes, and enhanced resistance to immunological depletion. The infographic was prepared based on Robert Chiesa et al., 2023 [61].

The modified cells were subsequently transduced with a lentiviral vector encoding a CAR recognizing CD7. This represents a highly elegant solution for T-ALL therapy. However, the approach requires more advanced manufacturing procedures than leukemias derived from B-cell precursors, since the target antigen must be preserved on the T lymphocytes used for CAR-T production, as the leukemia originates from these progenitor cells (Figure 4).

To date, no NK- or NKT-based products have received regulatory approval, with ongoing research confined to the clinical trial stage [62].

## 5. In Vivo CAR-T (CAR-X) Therapies

On top of that, CAR-T cells designed for the treatment of oncological and autoimmune diseases can now be prepared directly within the patient’s body (in vivo), a development made possible largely through advances in mRNA vaccine and viral vector technologies [63]. In contrast to classical cell therapy, in vivo CAR-T represents a hybrid modality at the interface of cell and gene therapy, in which endogenous immune cells are genetically reprogrammed in situ to express chimeric antigen receptors [64,65].

Compared with traditional ex vivo preparation, in vivo generation offers important advantages. Cells that are harvested from patients for in vitro modification often exhibit poor adaptability, particularly when obtained from individuals who are severely ill or have undergone intensive chemotherapy. The overall process of ex vivo CAR-T production is highly complex—requiring specialized laboratories, costly reagents, strict regulatory oversight, and significant time investment [66]. Consequently, the cost of ex vivo-manufactured CAR-T therapies remains extremely high [67]. One of the clear benefits of the in vivo approach is the elimination of both leukapheresis and lymphodepletion, procedures typically required before ex vivo therapy [68].

Mechanistically, in vivo CAR-T therapies rely on systemic or localized delivery of CAR-encoding genetic material using viral vectors (such as AAV or lentivirus) or non-viral carriers, including LNPs engineered for preferential uptake by lymphocytes [69]. Following delivery, resident T cells begin to express the CAR, undergo antigen-dependent activation, and expand within the patient, thereby generating a functional CAR-T population without external manipulation [70]. Preclinical studies have shown that in vivo–generated CAR-T cells can mediate potent and antigen-specific cytotoxic responses, particularly in CD19-targeted models, supporting the feasibility of this approach [71].

Nevertheless, while several in vivo gene therapies have already achieved FDA/EMA approval (Table 2), it remains at a preclinical/early clinical stage, highlighting the remaining delivery and safety difficulties. CAR-T and other CAR-modified immune cells (such as CAR-M, CAR-NK, or CAR-γδT) can be more extensively engineered in vitro than within the human body. Techniques such as CRISPR-based genome editing allow for the creation of “off-the-shelf” universal products, tailored for any recipient [72]. In addition, ex vivo manufacturing enables precise control over transgene copy number, integration site, and cell composition, features that remain difficult to regulate in in vivo approaches [73]. Safety mechanisms commonly used in ex vivo CAR-T products, such as suicide switches or stringent product quality control, are also more challenging to implement when CAR expression is induced directly within the patient [74].

Creating universal CAR-T cells remains an enormous challenge. However, growing knowledge of how to modify or neutralize genes responsible for graft rejection and graft-versus-host responses is steadily advancing the field—a crucial development for CAR-T therapies [75]. Such modifications are technically easier to perform in stem cells, yet differentiating genetically modified stem cells (iPSCs or HSCs) into mature lymphocytes remains a major obstacle [76]. Consequently, despite the higher susceptibility of stem cells to genetic manipulations, greater progress has been achieved in universalizing CAR-T cells derived directly from lymphocytes. Although genetic modifications in mature lymphocytes are less efficient than in stem cells, the practical difficulty of obtaining fully functional lymphocytes from stem cells often tips the balance in favor of the former.

This situation is somewhat analogous to the example discussed earlier in type 1 diabetes therapy: some research groups generate pancreatic cells from iPSCs or embryonic stem cells, while others focus on modifying cells harvested from deceased donors. The analogy lies in the question of whether it is easier to work with mature or stem cells. Stem cells divide indefinitely and are easier to genetically modify, but some target cell types are extremely difficult to derive from them. In such cases, modifying mature cells—despite their limited proliferative capacity—may be the more practical solution. Interestingly, CAR-NKT cells appear to have slightly greater proliferative potential than conventional T lymphocytes.

**Figure 5:**
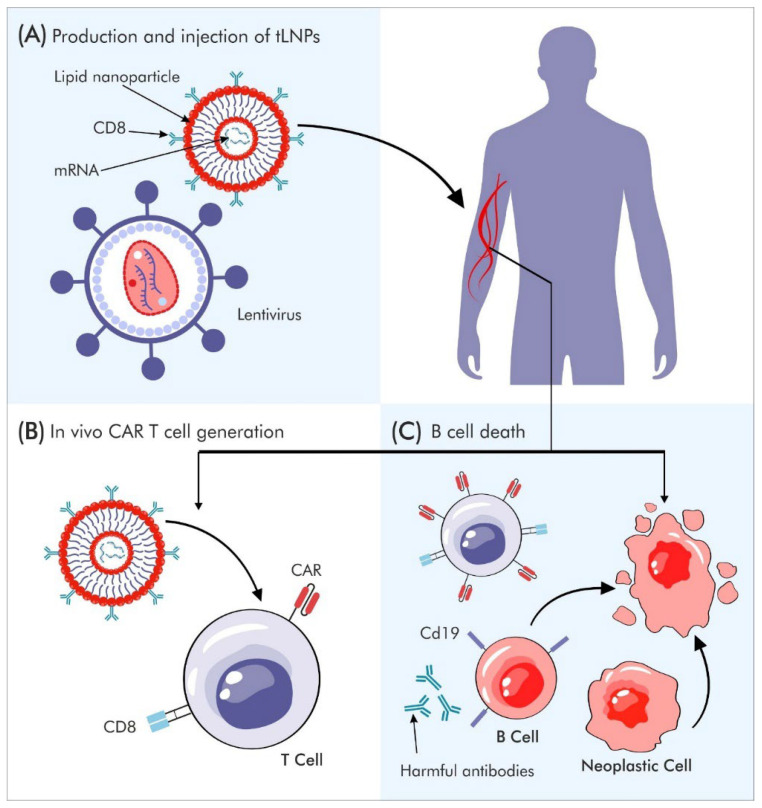
The generation of CAR-T cells and other CAR-engineered cell types is entering a new era. Some approaches focus on modifying T lymphocytes or the stem cells from which these lymphocytes can be produced ex vivo, using techniques such as CRISPR. Other strategies are carried out directly in vivo through the use of LNP technology or viral vectors. When the therapeutic target is CD19, lymphodepletion is not required because the same CAR that enables the destruction of CD19-positive malignant cells also mediates lymphodepletion.

There are, of course, many additional arguments for and against each approach. One important factor supporting in vivo strategies is the rapid development of lipid nanoparticle (LNP) technology following COVID-19 vaccination advances. Technology is now evolving into targeted LNPs (tLNPs) capable of directing delivery even to lymphocytes, facilitating in vivo therapeutic applications. As a result, CAR-T generation within the patient’s body is no longer a theoretical concept (Figure 5).

Redosing is another important consideration for CAR-T treatments. mRNA/LNP in vivo platforms may enable easier redosing, as LNPs can support repeated cycles of B-cell depletion with good tolerability, thereby enabling therapeutic cycles without repeatedly devastating the immune system. Allogeneic ex vivo platforms offer off-the-shelf advantages (e.g., rapid availability and standardized products); however, repeat dosing can be limited by alloimmunization and persistence issues. However, when the priority is clinician-directed control, allogeneic platforms incorporating switches and logic gates may provide a clear advantage in 2026 designs.

If, however, non-universal ex vivo CAR-T therapies are to compete with these in which CAR-T cells are generated in vivo, the latter may ultimately prove to be the winning strategy. The transgene encoding the CAR is now being integrated not only into classical immune cells. It has also been introduced, appropriately adapted or modified, into other cell types, such as astrocytes. These interventions were performed in vivo using a promoter that is active primarily in astrocytes (GfaABC1D). An AAV vector was used for delivery. This demonstrates that work on synthetic receptors is extending beyond the cell types traditionally associated with the immune system. CAR-X, in the subtitle, indicates that many different cell types are being targeted [13].

To contextualize CAR-based platforms within the broader regulatory landscape of gene and cell therapies, Table 2 summarizes FDA/EMA-approved products.

## 6. Comparative Evaluation: Ex Vivo and In Vivo Gene Modification Strategies

### 6.1. Vector Safety: Ex Vivo vs. In Vivo Approaches

Vector safety remains one of the major challenges in gene and cell therapies, with risks and mitigation strategies differing substantially between ex vivo and in vivo approaches. In ex vivo therapies (e.g., modified hematopoietic stem cells, CAR-T/CAR-NK cells, and iPSC-derived cells), lentiviral and retroviral vectors—which integrate into the genome—are predominantly used. The principal risk associated with these vectors is insertional mutagenesis leading to proto-oncogene activation. This risk was particularly evident in early generations of gene therapies, such as the development of leukemia in ADA-SCID trials.

Contemporary solutions have markedly improved safety, most notably through the use of self-inactivating (SIN) lentiviral vectors. These improvements have been achieved, for example, by incorporating chromatin insulators (e.g., cHS4) [77], employing weaker promoters (such as PGK instead of MSCV), and limiting vector copy number. In addition, CAR-T therapies commonly incorporate so-called safety switches (e.g., tEGFR, iCasp9, RQR8), which enable selective elimination of the modified cells in the event of toxicity or oncogenic transformation [10,58].

A different situation applies when induced pluripotent stem cells (iPSCs), rather than lymphocytes or HSCs, are used. In this case, initial reprogramming typically relies on episomal vectors, after which the introduction of therapeutic transgenes can be achieved using a variety of alternative delivery systems.

In in vivo therapies, the risk of genomic integration must be minimized; therefore, episomal vectors such as adeno-associated viruses (AAVs) are commonly used. However, this approach introduces other challenges, including strong immune responses against the AAV capsid—particularly the presence of neutralizing antibodies—as well as potential immunogenicity of the newly expressed transgene product. Strategies to improve safety include AAV capsid detargeting, for example, through surface modifications that reduce immune recognition [78].

Lipid nanoparticle (LNP)–delivered mRNA, which enables transient expression, offers an even lower risk of permanent genomic alteration and generally exhibits reduced immunogenicity. A remaining challenge for LNP-based approaches is precise control of tissue tropism; however, as demonstrated in the context of hypercholesterolemia, this is increasingly becoming feasible [47].

### 6.2. Durability of Expression: Ex Vivo vs. In Vivo

The durability of therapeutic expression depends primarily on whether the vector integrates into the genome—allowing long-term expression in dividing cells—or remains episomal, which leads to loss of expression in proliferating tissues. When genome editing is performed, however, the situation changes fundamentally: the genetic modification is maintained for as long as the edited cell population persists.

In ex vivo therapies, lentiviral vectors provide stable, long-term expression lasting many years, even after transplantation (e.g., Zynteglo, Casgevy, CAR-T therapies), as the modified hematopoietic stem cells or lymphocytes divide and transmit the integrated transgene to their progeny. In in vivo therapies, AAV vectors support durable expression in post-mitotic cells—such as neurons, hepatocytes, muscle cells, and auditory sensory cells—as demonstrated by therapies, including Zolgensma for spinal muscular atrophy, Luxturna, and otoferlin gene therapy [12].

However, in proliferating tissues (e.g., hepatocytes in pediatric patients), expression declines over time due to dilution of episomal vectors. LNP-delivered mRNA enables transient expression lasting weeks to months, which is advantageous for in vivo CAR-T approaches by avoiding permanent genomic alterations [35], but represents a limitation for diseases that require sustained gene expression. Strategies to improve durability include the use of integrating lentiviral vectors in vivo (rarely employed because of safety concerns), episomal amplification systems within AAV vectors, or repeated low-dose administration of LNPs. As noted above, the situation differs when LNPs are used to deliver gene-editing systems, as the therapeutic effect can persist despite the transient presence of the editor. LNPs, similar to viral vectors, can be engaged in the targeted delivery [47].

An instructive comparison in this context is provided by gene editing approaches targeting tRNA genes in the treatment of nonsense mutations [8]. Earlier strategies attempted to introduce a modified tRNA transgene capable of recognizing stop codons and inserting an amino acid during translation. In such cases, when delivered by AAV, the therapeutic effect was transient because episomal AAV genomes were gradually lost.

By contrast, current approaches use AAV to deliver a gene-editing system that modifies the endogenous tRNA gene. In this scenario, the AAV vector performs its function before being lost, while the corrected genomic sequence—and thus the therapeutic effect—persists. Lentiviral vectors carrying tRNA transgenes previously offered more durable expression; however, their random genomic integration. The associated risk of insertional mutagenesis was considered unacceptably high, and progress in eliminating those effects seems to be still inefficient [79].

In summary, AAV-mediated delivery of a tRNA transgene resulted in a transient effect, whereas lentiviral delivery provided durability at the cost of increased oncogenic risk. The newer strategy resolves this trade-off by using AAV not to introduce a tRNA transgene, but to install a permanent genomic modification of the endogenous tRNA gene—so that even after the vector is lost, the therapeutic benefit remains.

### 6.3. Tissue and Cellular Tropism

Tissue and cellular tropism can be achieved through several distinct mechanisms and depend strongly on whether the therapy is performed ex vivo or in vivo. In ex vivo therapies, the concept of “tropism” is largely replaced by cell selection and directed delivery, as the genetic modification is performed outside the body. Typically, patient cells (hematopoietic stem cells (HSCs), keratinocytes, fibroblasts, or pancreatic cells) are isolated; then a therapeutic transgene is introduced, or a pathogenic mutation is edited, and the modified cells are returned to the patient. iPSCs are usually generated and expanded ex vivo, and clinically relevant, genetically modified derivatives are transplanted rather than iPSCs themselves.

For stable transgene expression in dividing cells, lentiviral or retroviral vectors are most commonly used because they integrate into the genome, supporting long-term expression. A representative example, discussed in Section 2, is therapy for sickle cell disease and β-thalassemia, in which HSCs are transduced with a lentiviral vector carrying a modified β-globin gene under specific regulatory control (β-globin promoter and locus control region), followed by transplantation after myeloablation. Similarly, in dystrophic epidermolysis bullosa, keratinocytes and fibroblasts are retrovirally modified with cDNA of the COL7A1 gene and applied as a graft directly to the wound site, which achieves anatomical targeting through the surgical procedure [34]. Sometimes, full transgene insertion is not required, but CRISPR-based editing, base editing, or prime editing could be performed directly in isolated cells to correct specific mutations (editing of NCF1 in chronic granulomatous disease) [9]. In different instances, in type 1 diabetes, hypoimmune donor-derived β-cells engineered by B2M and CIITA knockout combined with CD47 overexpression were transplanted into skeletal muscle to enable persistence and insulin production without immunosuppression [11]. Although this approach can be clinically meaningful, it should be underscored that anatomical delivery to the pancreas, despite being technically more challenging, may offer superior engraftment and functional integration [80,81].

Overall, in ex vivo therapy, the critical determinants of “targeting” are precise selection of the starting cell population and directed transplantation to the desired anatomical compartment (bone marrow, skin, muscle, or CNS), rather than vector tropism.

On the other hand, in in vivo therapies, tropism returns to its classical meaning: therapeutic transgenes or genome-editing systems are delivered directly into the body, requiring strong vector tropism and engineered targeting strategies. For many inherited liver diseases, in vivo delivery is more favorable than for CNS disorders because intravenously administered lipid nanoparticles (LNPs) naturally accumulate in the liver and are efficiently taken up by hepatocytes. This was used, for example, in CPS1 deficiency, where LNPs deliver mRNA encoding an adenine base editor together with a guide RNA to enable in vivo editing [82]. Despite the intrinsic liver tropism of LNPs, additional engineering can further improve selectivity. In hypercholesterolemia, in vivo editing of ANGPTL3 has been enhanced by LNP modifications that promote interactions with LDL receptor-related uptake pathways and scavenger receptors on hepatocytes. This strategy partially mimics physiological LDL clearance from blood, thereby increasing liver-selective delivery [47]. In contrast, in Huntington’s disease, an AAV vector encoding an artificial microRNA targeting HTT (miHTT) was administered directly into the striatum using MRI-guided convection-enhanced delivery, leveraging neuronal tropism of AAV while still relying on local administration to overcome the blood–brain barrier [51]. In congenital deafness due to OTOF mutations, a dual-AAV system carrying OTOF cDNA under a Myo15 promoter can drive expression in cochlear hair cells. However, the vector is still delivered locally to the inner ear via otolaryngologic surgery [12].

In emerging in vivo CAR strategies, lipid nanoparticles with CAR mRNA exploit tropism for T or NK lymphocytes.

To sum up, in ex vivo therapies, “tropism” is effectively implemented through cell-type selection (HSCs, iPSC-derived products, keratinocytes) combined with targeted transplantation to the appropriate site (bone marrow, skin, muscle, or CNS). In in vivo therapies, the dominant determinants are natural or engineered vector tropism (AAV for neurons and hair cells; LNPs for hepatocytes, including additional LDLR/scavenger-mediated uptake), tissue-restricted promoters, and local administration strategies when systemic delivery is inadequate (MRI-guided intracranial infusion or inner-ear injection). Altogether, tropism, promoter specificity, delivery route, and in some settings hypoimmune engineering enable efficient delivery to selected tissues while minimizing dose requirements and safety risks (Table 3).

### 6.4. Tunability of Expression: Ex Vivo vs. In Vivo

Tunability—the control of expression level, timing, and tissue specificity—is critical for minimizing toxicity and optimizing therapeutic efficacy, but the available strategies differ substantially between ex vivo and in vivo approaches. In ex vivo therapies, control is comparatively straightforward: strong constitutive promoters (e.g., EF1α, PGK, MSCV) can be used in combination with safety switches (such as tEGFR or iCasp9), or additional regulatory elements can be incorporated, including miRNA-responsive circuits in next-generation CAR designs. Representative examples include hypoimmunogenic CAR-T and CAR-NK therapies [10,58] (Table 4).

In in vivo therapies, tunability is more challenging. Using AAV or LNP platforms, it is difficult to introduce the number and complexity of regulatory elements required for features such as safety switches (e.g., tEGFR). In this context, tissue-specific promoters can provide partial solutions for certain patients by restricting expression to target cell types, such as Myo15 in otoferlin-related deafness [12], GfaABC1D in astrocytes [64], or thyroxine-binding globulin (TBG) in hepatocytes [83].

The transient activity of LNP-delivered mRNA provides an inherent form of temporal tunability through short-lived expression, which is advantageous in vivo CAR-T approaches [35] and personalized gene editing strategy [82] (Table 5).

## 7. Summary

In recent years, a breakthrough has occurred across several types of biological therapies that had previously faced significant challenges—most notably in gene therapies and various forms of cell therapies.

This progress results from several key factors. Among them is the improvement of cell differentiation techniques, which has expanded the range of cell types that can be generated for therapeutic use. Equally important are the advances stemming from CRISPR technology and its derivatives, which have revolutionized the precision and scope of genetic modification.

Another crucial factor has been the enhancement of transgene delivery systems, driven by progress in both viral vector design and lipid nanoparticle (LNP) technology—the latter accelerated by innovations developed during COVID-19 vaccination programs.

Nevertheless, therapies targeting blood cells and the liver remain the most accessible. The former are easier to modify ex vivo, whereas the latter are a natural target for LNPs. Researchers are demonstrating increasing ingenuity: a wide spectrum of genome-editing strategies is being explored, extending even to Cas13, which degrades aberrantly spliced mRNA. CRISPR-based genome editing is not always the optimal solution. In some cases, such as otoferlin deficiency, the introduction of a single transgene enables therapy across many different mutations. Conversely, editing the gene encoding tRNA may offer improvements in numerous rare diseases. Vectors do not necessarily need to deliver mRNA; they may instead deliver miRNA, as in Huntington’s disease. Moreover, research teams are modifying promoters: for example, the Myo15 promoter for congenital deafness, the nestin promoter in viral therapies for glioblastoma, or GfaABC1D when introducing a CAR transgene in vivo into astrocytes.

The size of the gene is a very important issue when transgenes are applied. Taken together, these approaches show that current efforts fall increasingly within the domain of synthetic biology rather than traditional genetic engineering.

Even more significant advancements can be observed in CAR-T therapy and its variants, where improved understanding and engineering have led to major therapeutic gains.

Across all these developments, the foundation of success lies in the deepening understanding of disease mechanisms and cellular biology, which continues to shape and guide the evolution of next-generation therapies.

An overview of the generation of engineered cellular therapies derived from autologous and allogeneic donors is presented in Table 6.

The examples of therapies described in this work are grouped according to the organs or structures they target. Table 7 illustrates, in the context of specific diseases, which organ-directed therapeutic strategies have progressed most rapidly in recent years. This does not imply that other approaches are not being developed; rather, it highlights where the fastest advances have occurred.

## Figures and Tables

**Table 1:** Representation of genes mentioned in the article based on their molecular size and the tested therapeutic approach.

Gene/Disease/Therapy	cDNA/ORF Length (Approx., bp)	Notes/Delivery Technology Used in the Publication or Context	“Long Gene”?	Source in Publication or Context
OTOFERLIN (OTOF)/congenital deafness	~5994 bp (ORF)	Large gene requires two cooperating AAV vectors (dual AAV) with split-intein/ splicing strategy (AP-SD/AP-SA) to enable recombination	Yes	Section 3, Figure 3C
COL7A1/recessive dystrophic epidermolysis bullosa (RDEB)	~8832 bp (ORF)	Retroviral vector delivering full-length COL7A1 cDNA; retro/lentiviral vectors tolerate >8 kb payloads	Yes	Section 2, Figure 1C
WAS (Wiskott–Aldrich syndrome)	~1506 bp (ORF)	Lentiviral vector carrying WAS cDNA (small gene; no packaging constraint)	No	Section 2
ATP1A3/alternating hemiplegia of childhood (AHC)	~3039 bp (ORF)	AAV9-mediated mutation correction; fits within a single AAV vector	No	Section 3
LMNA (progerin mRNA)/Hutchinson–Gilford progeria syndrome	~1989 bp (ORF)	Lentiviral Cas13 + gRNA approach; no full-length LMNA transgene—selective degradation of mutant mRNA (small construct)	No	Section 3, Figure 3B
HTT (huntingtin)/ Huntington’s disease	~9432 bp (ORF)	AAV delivering artificial miRNA (miHTT); small miRNA cassette, not full-length HTT cDNA	No (miRNA only)	Section 3, Figure 3A
CPS1/carbamoyl phosphate synthetase I deficiency	~4500 bp (ORF)	In vivo adenine base editing (ABE) via LNP; mutation correction without introduction of a full transgene	No	Section 3, Figure 3D
HBB (β-globin)/sickle cell disease, β-thalassemia	~441 bp (ORF)	Lentiviral delivery of modified βT87Q-globin (very small gene)	No	Section 2
NCF1 (p47phox)/chronic granulomatous disease	~1170 bp (ORF)	ex vivo prime editing of NCF1 mutation; gene correction without transgene insertion	No	Section 2, Figure 1B
CSF1R/adult-onset leukoencephalopathy (ALSP)	~2916 bp (ORF)	Microglia transplantation or CRISPR-based editing; no CSF1R transgene delivery	No	Section 2
DMD (dystrophin)/ Duchenne muscular dystrophy	Full dystrophin: ~11,000–14,000 bp (ORF); micro-dystrophin: ~3500–4400 bp	Full gene too large for AAV → truncated micro-dystrophin constructs compatible with single AAV (e.g., Elevidys, Sarepta)	Yes (full gene)	Section 3 (added as classic long-gene therapy example)

**Table 2:** Genetic and cell therapies currently approved by the FDA and EMA.

Product (Trade Name)	INN/Genetic Construct	Therapy Type	Indication	Regulatory Agency
Luxturna^®^	Voretigene neparvovec-rzyl	In vivo gene therapy (AAV)	Leber congenital amaurosis (RPE65 mutation)	FDA, EMA
Zolgensma^®^	Onasemnogene abeparvovec	In vivo gene therapy (AAV)	Spinal muscular atrophy (SMA)	FDA, EMA
Hemgenix^®^	Etranacogene dezaparvovec	In vivo gene therapy (AAV)	Hemophilia B	FDA, EMA
Beqvez™	Fidanacogene elaparvovec-dzkt	In vivo gene therapy (AAV)	Hemophilia B	FDA
Upstaza™	Eladocagene exuparvovec	In vivo gene therapy (AAV)	Aromatic L-amino acid decarboxylase (AADC) deficiency	EMA
Adstiladrin^®^	Nadofaragene firadenovec	In vivo gene therapy (adenovirus)	Non-muscle invasive bladder cancer	FDA
Vyjuvek™	Beremagene geperpavec	In vivo gene therapy (HSV-1 vector)	Dystrophic epidermolysis bullosa	FDA
Imlygic^®^	Talimogene laherparepvec	Oncolytic virus (GM-CSF expressing HSV-1)	Melanoma	FDA, EMA
Strimvelis^®^	Autologous CD34^+^ cells with the ADA gene	Ex vivo gene therapy	ADA-SCID	EMA
Zynteglo^®^	Betibeglogene autotemcel	Ex vivo gene therapy	β-thalassemia	FDA, EMA
Lyfgenia^®^	Lovotibeglogene autotemcel	Ex vivo gene therapy	Sickle cell disease	FDA
Casgevy^®^	Exagamglogene autotemcel (exa-cel)	CRISPR-edited autologous HSCs	Sickle cell disease, β-thalassemia	FDA, EMA
Kymriah^®^	Tisagenlecleucel	CAR-T cell therapy	B-cell acute lymphoblastic leukemia, lymphomas	FDA, EMA
Yescarta^®^	Axicabtagene ciloleucel	CAR-T cell therapy	B-cell lymphomas	FDA, EMA
Breyanzi^®^	Lisocabtagene maraleucel	CAR-T cell therapy	B-cell lymphomas	FDA
Abecma^®^	Idecabtagene vicleucel	CAR-T cell therapy	Multiple myeloma	FDA, EMA
Carvykti^®^	Ciltacabtagene autoleucel	CAR-T cell therapy	Multiple myeloma	FDA, EMA
Tecelra™	Afamitresgene autoleucel	Genetically modified T cells (TCR-T)	Synovial sarcoma	FDA

**Table 3:** Addressing different aspects of tissues and cells targeting strategies.

Strategy/ Technology	Type of Therapy (Ex Vivo/In Vivo)	Target Cell/ Organ	Examples from Publications (Manuscript)	Advantages/Expected Impact on Low Dose/Safety
Ligand-modified/targeted LNPs (LDLR/ scavenger)	in vivo	Hepatocytes (liver)	LNPs tuned to bind LDLR and scavenger receptors, mimicking LDL uptake [47]	Very high liver selectivity lower required dose, reduced extrahepatic exposure
Tissue-specific promoters	ex vivo and in vivo	Hair cells (inner ear), astrocytes (CNS), erythroid cells, hepatocytes	Myo15 (otoferlin) [12], GfaABC1D (astrocyte CAR in astrocytes) [64], TBG (liver)	Major dose reduction by avoiding off-target expression
Ligand-modified/targeted LNPs (general)	in vivo	Hepatocytes; lymphocytes/T/NK cells	LNPs with LDLR or scavenger-receptor ligands [47]	5–50× dose reduction, lower systemic toxicity
Vector tropism + local administration	in vivo	Striatum (CNS), inner ear	AAV9 + convection-enhanced delivery (Huntington’s) [51]; local AAV delivery (inner ear) [12]	Drastic reduction in systemic dose

**Table 4:** Multi-gene enhancement and tunability.

Number of Genes/ Modifications	Therapy Type	Examples of Modifications in a Single Vector/Cell	Current Status (2026)	Benefits (Including Low-Dose/Tunability)	Examples from Publications or Trends (Manuscript)
4–5 modifications	ex vivo CAR	CAR + TRAC KO + B2M KO + CIITA KO + CD47 high level	Approved/late clinical	Increased persistence, improved safety at lower cell doses	Hypoimmune CAR-T [58]
3 modifications	ex vivo hypoimmune islets (type 1 diabetes)	B2M KO + CIITA KO + CD47 high level (lentiviral)	Clinical (first patient)	Allogeneic universality, reduced immunosuppression, lower cell dose	Carlsson et al., 2025 (allogeneic beta cells) [11]
8–9 modifications	ex vivo iPSC-derived	3 KO + 4 native transgenes + 2 synthetic CAR (dual)s	First patients	Full universality + controlled activation	iPSC-CAR-NK in scleroderma [10]
Multi-editing (2–4 loci)	in vivo base/prime editing	Single-dose editing of multiple mutations or genes (e.g., prime editing, tRNA suppressor)	Preclinical/early	One-time dosing addressing multiple defects	Pierce et al., 2025 [8]

**Table 5:** Tunability based on regulation of expression.

Level of Tunability	Technology/Regulatory Element	Type of Therapy	Current Status (2026)	Examples/ Potential Applications	Benefit for Low Dose/Safety
Constitutive (fixed)	Strong Pol II promoters (CAG, Cβ, PGK-like)	ex vivo and in vivo	Approved/clinical	Otoferlin [12], CAR in astrocytes [64], β-globin LCR (Zynteglo)	No tunability higher dose may be required and carries increased risk
Tissue-specific	Tissue-specific promoters (Myo15, GfaABC1D, TBG)	ex vivo and in vivo	Approved/early clinical	Myo15 in otoferlin therapy [12], GfaABC1D in astrocyte CAR [64], TBG in liver	Dose reduction by 1–2 orders of magnitude by avoiding off-target expression
Constitutive transgene with miRNA regulation	Pol II promoter + miRNA scaffold (e.g., miR-451-like)	in vivo	Clinical (phase 2/3)	Artificial miRNA (mHTT) in Huntington’s disease (AMT-130); constitutive Pol II + miRNA scaffold [51]	Continuous miRNA production in striatal neurons; control mainly via local AAV delivery
Transient (short-term)	LNP–mRNA, transient base editing	in vivo	Clinical (e.g., CPS1); in vivo CAR	In vivo CAR-T [35], personalized CPS1 editing [82]	Lowest dose, no integration, expression lasting weeks to months
Conditional/logic-gated	SynNotch circuits, miRNA-responsive logic gates	ex vivo (CAR)	Preclinical (2024–2025)	CAR-T with antigen sensing + miRNA + OFF-switch (emerging)	Very low effective dose and reduced toxicity via conditional expression

**Table 6:** Sources and manufacturing strategies of autologous and allogeneic engineered cell therapies.

Cell Type/Feature	Number of Possible Modifications	Difficulty of Obtaining the Cells	Cost	Use
Universal/ Autologous, in vivo	Limited	Intermediate at present	Lowest	Clinical trials
Universal, ex vivo	Very high. In practice, CAR-NK or CAR-NKT rather than CAR-T	Highest differentiation can be complex	Ultimately lower than for autologous cells	Clinical trials
Autologous, ex vivo	Limited, but broader in in vivo approaches	Currently the lowest	High	Clinical use and clinical trials

**Table 7:** Organ-targeted therapeutic approaches with the fastest recent progress in selected diseases.

Organ/Method	Cells Modified Ex Vivo with Viral Vectors in This Paper	Viral Vectors In Vivo	LNP In Vivo
Brain	YES—microglia; universal engineered cells	AAV	NO
Liver	NO	Mainly LNP	YES—rapid progress
Blood/Bone marrow	YES, e.g., HSCs (CD34^+^)	CAR-T in vivo	CAR-T in vivo
Heart	YES	NOT in this paper	NOT in this paper
Pancreas	YES—after differentiation of ESCs; also CRISPR-modified cells from deceased donors	NOT in this paper	NOT in this paper
Skin	YES	NOT in this paper	NOT in this paper
Systemic	NOT in this paper	One example: postnatal progeria (animal studies), lentiviral	NOT in this paper
Embryonic stage	PROHIBITED—modification banned, but selection of embryos allowed if genetic testing performed early	PROHIBITED	PROHIBITED

## Data Availability

Not applicable.

## Abbreviations

The following abbreviations are used in this manuscript:

AAV	adeno-associated virus
ABE	adenine base editing
AHC	alternating hemiplegia of childhood
ALSP	adult-onset leukoencephalopathy with axonal spheroids and pigmented glia
B2M	beta-2 microglobulin
CAR	chimeric antigen receptor
CAR-NK	chimeric antigen receptor natural killer
CAR-NKT	chimeric antigen receptor-natural killer T-cell
CAR-T	chimeric antigen receptor T-cell
CGD	chronic granulomatous disease
CIITA	class II major histocompatibility complex transactivator
CNS	central nervous system
COL7A1	gene encoding the alpha chain of type VII collagen
CPS1	carbamoyl phosphate synthetase 1
CRISPR	clustered regularly interspaced short palindromic repeats
CSF1R	colony-stimulating factor 1 receptor
FDA	Food and Drug Administration
HGPS	Hutchinson-Gilford progeria syndrome
HIP	hypoimmune
HLA	human leukocyte antigens
HSCs	hematopoietic stem cells
iPSCs	induced pluripotent stem cells
LDL	low-density lipoprotein
LMNA	lamin A
LNPs	lipid nanoparticles
NK	natural killer
PAM	protospacer adjacent motif
RDEB	recessive dystrophic epidermolysis bullosa
tLNPs	targeted lipid nanoparticles
Treg	regulatory T-cells
WAS	Wiskott–Aldrich
